# Identification of struggling readers or at risk of reading difficulties with one-minute fluency measures

**DOI:** 10.1186/s41155-021-00174-z

**Published:** 2021-03-20

**Authors:** Maíra Anelli Martins, Simone Aparecida Capellini

**Affiliations:** 1 Speech and Hearing Sciences Department, Sao Paulo State University “Júlio de Mesquita Filho”, UNESP, Campus Universitário, Av. Hygino Muzzi Filho, 737, 17525-900 Marília, SP Brasil; 2 São Paulo State University “Júlio de Mesquita Filho”, UNESP, Investigation Learning Disabilities Laboratory (LIDA), Marília, SP Brazil; 3 “Language, Learning, Education” CNPq Research Group, Marília, SP Brazil; 4 Full Professor of Speech and Hearing Sciences Department, São Paulo State University “Júlio de Mesquita Filho”, UNESP, Marília, SP Brazil

**Keywords:** Struggling readers, Universal screening, Academic screening, Academic difficulties, Oral reading fluency, Reading research

## Abstract

**Background:**

To identify readers who are struggling or at risk of reading difficulties, reference standards in oral reading fluency (ORF) are used to conduct an assessment that is based on a widely reported method known as curriculum-based measurement (CBM), which itself is based on 1-min fluency measures. The purpose of this study was to evaluate students’ ORF (with a 1-min fluency measure) to characterize their fluency and to determine references of appropriate development in reading at the 50th percentile.

**Method:**

For this study, a database of readings made available by the Learning Studies Research Laboratory was used. This database consisted of 365 readings by elementary-school students from the third to fifth grades in two cities in the interior of the state of São Paulo from two different public school systems that use the same teaching methodology. The data consisted of digital audio recordings of the passage “The Umbrella” (text suitable for schooling levels) of the Protocol for Assessment of Reading Comprehension procedure. For this procedure, three steps were performed: step 1—listening to the 365 readings and assessing the scores for the number of words read correctly per minute; step 2—the calculation of the mean and percentiles for each grade; and step 3—the adaptation of the reference table to indicate students eligible to receive reading fluency intervention.

**Results:**

Third-year students who correctly read 86 or more words per minute, fourth-year students who correctly read 104 or more words per minute, and fifth-year students who correctly read 117 or more words per minute were considered students who had made adequate progress in reading.

**Conclusion:**

It was possible to classify students based on the 1-min fluency measures, with reference intervals of words read correctly per minute per school year (for the third, fourth, and fifth years) for those who were making adequate progress in reading and reference intervals for those who were considered readers who were struggling or at risk of reading difficulties.

## Background

Little research has been conducted in Brazil on measures to assess reading fluency (Gentilini et al, 2020; Andrade, Celeste, & Alves, 2019; Moutinho, 2016; Pacheco & Santos, 2017; Peres & Mousinho, 2017), and a search for research on reading fluency in official documents of the Brazilian Ministry of Education (Martins, 2018) also reveals that such measures are not a type of assessment that is widely known or applied by teachers within the classroom. Nonetheless, research has continually indicated the importance of developing oral reading fluency (ORF; reading with appropriate rate, accuracy, and prosody) as a vital and necessary skill for the overall development of proficient reading (Machado, Santos, & Cruz, 2019; Rasinski & Young, 2017).

In addition to the lack of Brazilian research widely exploring this theme, the low performance data of Brazilian students in reading indicates that these students also face difficulties in learning this highly complex activity, including the many who do not become proficient, effective readers. It is noted that this is a recurring problem that affects students and, consequently, concerns educators. As is clear from the evaluations conducted throughout the national territory (large-scale evaluations), the problem has continued throughout the years and affects even the regions with the best educational indexes or socioeconomic status.

### Measures assessment of reading oral fluency

The method widely publicized as curriculum-based measurement (CBM) is a curriculum-based progress-monitoring method for measuring growth in specific areas of basic knowledge and skills and assessing the effects of instructional programs (response to intervention). Curriculum-based assessment, as a longstanding assessment practice asserting that learning assessments should be based on what has been taught, has become popular in the field of special education. Thus, the CBM method is described as curriculum-based, as it is used within the context of the school curriculum (Deno, 1985).

The CBM method proposes simple measures for the assessment of academic competence that can be applied quickly by teachers. These measures help provide an overview of each student’s academic development; furthermore, when these simple measures are applied systematically over time, they can be used to track a student’s potential difficulties (Fuchs, 2017).

For example, to identify struggling readers, reference standards for ORF are used, which, based on the CBM assessment method initially proposed by Deno (1985), enable reading analysis in just 1 min (e.g., the number of words read correctly per minute–WCPM). The most widely used assessment of ORF, which focuses on two of the three components of fluency (rate and accuracy), simply requires the student to read a grade-appropriate passage, which they have not seen previously, for 1 min. At the end of 1 min, errors are subtracted from the total words read, and then the WCPM score is calculated (Hasbrouck & Tindal, 2006).

Thus, the method was developed to create procedures for measuring progressive development in a simple, reliable, and valid way. These procedures enable teachers to frequently and repeatedly measure students’ progress in basic reading, spelling, writing, and expression skills (Rasinski, 2004).

Regarding reading fluency assessment, it is recommended that the scoring of the number of words read correctly per minute (WCPM) and the number of words read incorrectly per minute (WIPM) be performed with three passages of the same difficulty level to then calculate the mean score. Thus, the WCPM measure can serve to screen for academically at-risk students, assign placement in remedial and special education programs, monitor student progress, improve teaching programs, and predict performance in high-risk assessments (Hasbrouck & Tindal, 2006; Rasinski, 2004).

A series of discussions began in the last decade in Brazil on the question of the “wait to fail to act” model, which highlighted the importance of the early identification of learning difficulties. There are also discussions about the broadening of knowledge about the advantages of early identification and scientific evidence-based assessment and screening methods (Almeida, Piza, Toledo, Cardoso, & Miranda, 2016; Batista & Pestun, 2019; Brito, Seabra, & Macedo, 2018; Justi & Cunha, 2016; Mayeda, Navatta, & Miotto, 2018; Nicolau & Navas, 2015; Palles da Silva & Guaresi, 2019; Rodrigues & Ciasca, 2016; Silva & Capellini, 2017; Silva & Capellini, 2019a; Silva & Crenitte, 2016).

According to Elliott, Huai and Roach (2007), several factors contribute to the prevalence of the “wait to fail to act” model, such as the fact that educators understand that there is a certain heterogeneity of development and learning among students and seek to allow appropriate time for this development. By doing so, they are also allowing students a fair chance of progressing without early determination of the problem. Another factor for the prevalence of this action model is the fact that few large-scale screening instruments are time efficient and technically simple for teachers to apply.

In the Brazilian literature, early screening instruments are recent and focus primarily on metalinguistic skills, such as the “Early Identification and Reading Problems Protocol” (Capellini, César, & Germano, 2017), the “Evaluation of Cognitive-Language Skills Protocol: Professional and Teacher’s Book” (Capellini, Smythe, Silva, 2017) and the “Protocol for Cognitive-Language Skills Assessment of Students in Early Literacy” (Silva & Capellini, 2019b). These instruments assess skills considered predictive of literacy, such as reading and writing skills; arithmetic; auditory and visual processing; metalinguistic skills; and processing speed with the rapid automatic naming test. Some tests evaluate mathematical logical reasoning, for example, the “Cognitive-Language Skills Assessment Protocol.”

Likewise, there has been a movement in Brazilian research in recent years to describe the importance of reading fluency measures, especially those related to using a chronometer for timing as measures for screening difficulties, in addition to the development of instruments to assist in this assessment. Alves et al. (2019) described such issues in the most recent publication of the LEPIC® software, which proposes a semiautomatic and instantaneous reading fluency analysis to assess and assist in diagnostics or to monitor reading skills. This analysis focuses on the importance of evaluating parameter fluency, which may include indicators of reading problems such as dyslexia. Another instrument recently developed by Brazilian researchers is a collection of passages in sequential order according to difficulty level and suitable for elementary-school students from the first through fourth grades, called the “Reading Fluency Performance Assessment” (Martins & Capellini, 2018).

Additionally, on 22 February 2018, the More Literacy Program (PMAlfa) was created via MEC Ordinance No. 142, a strategy by the Ministry of Education that aims to strengthen and support school units in the process of increasing the literacy of elementary-school students enrolled in the first and second grades; the program fulfills the criteria established in the Common National Curriculum Base (CNCB). The objective of the program is to perform reading, writing, and math evaluations. For the first time, a formal program of the Brazilian government will evaluate the fluency and accuracy in the reading ability of students in the second grade of elementary school. The assessment is performed individually and uses a proprietary application suitable for smartphones or tablets.

However, despite efforts to create adequate assessment procedures for ORF, research into the characterization of ORF in this population is still incipient. Pacheco and Santos (2017), for example, evaluated three groups of readers in relation to reading fluency who were classified into three groups: group I–second-grade readers with little reading experience and expectation of low reading fluency; group II–second-year high school readers with the expectation of having slightly more reading experience and moderate fluency; and group III–readers with a higher education level. However, the relatively small sample consisted of 12 participants (four participants in each group), and the reading rate was evaluated by using the number of words read compared to the total reading time measured in seconds, considering a total reading time of 180 s (3 min).

In another study (Moutinho, 2016), 46 sixth-grade students from public and private schools were evaluated by measuring the WCPM in 1 min from three different passages. However, the article focused on describing the accuracy errors, i.e., the number and type of WIPM, while data for the WCPM are not presented. Other researchers evaluated 55 students from the third to the seventh grades with the number of words per minute, reading four different types of passages, and analyzing student performance in each (Dellisa & Navas, 2013).

Some researchers have also conducted reading fluency assessment with elementary students, as in a study that evaluated 32 students in ninth grade and calculated the speed of words read per minute (using the formula of total number of words from the passage, divided by the time in seconds spent to complete the reading, and multiplied by 60) (Komeno, Ávila, Cintra, & Schoen, 2015). Furthermore, in another recent study, researchers characterized the ORF by 232 middle-grade students from the sixth to the ninth grades from public and private education. The study provided an estimate of the expected values for each grade surveyed by reading an easy passage based on the 1-min oral fluency assessment, with scores for words read per minute and WCPM (Andrade et al., 2019).

While only a small number of studies for elementary and middle students exist, even fewer studies evaluate reading fluency in high school students or adults. One research study evaluated 88 students in the second grade of high school. The CBM method was followed by selecting a passage compatible with students’ age and grade and comprising subjects corresponding to the basic curriculum studied in the classroom. Students read three different passages, lasting 1 min each, for the subsequent calculation of the number of WCPM (Oliveira, Amaral, & Picanço, 2013). Only one study evaluating reading fluency in adults was found, in which the sample consisted of 30 adolescents and adults who were evaluated by measuring the number of words per minute (Peres & Mousinho, 2017).

The assessment of ORF conducted through WCPM scores presents 30 years of validation research indicating that this is a valid and reliable measure that reflects a student's overall performance in reading development during the first years after literacy (Morris et al., 2017a, b; Tindal, 2017; Valencia et al., 2010). Reading fluency benchmarks have been used both for screening and for monitoring reading development, and research in these fields seeks to answer questions such as “How is student performance compared to their peers?” and “Who are the students struggling with reading?” This practice of frequent assessment enables early intervention and the planning of activities that focus on the skills already acquired and those that still require further attention.

Benchmarks in ORF have been established by American researchers and collected from a range of students, from those identified as talented or otherwise exceptionally skilled to those diagnosed with reading disabilities, such as dyslexia. The largest sample of the ORF benchmark was collected from schools and districts in 23 states in the USA for over 4 years. Based on their vast experience in interpreting ORF data, it was established that a score of 10 words above or below the 50th percentile should be interpreted as an expected score, meaning that students are making satisfactory reading progress (Hasbrouck & Tindal, 2006).

Given the implications that ORF benchmarks would have for Brazilian education, a study to determine a fluency reference through appropriate assessment material would be of great relevance. This benchmarking considers the indication of a median score (50th percentile), with scores of 10 words above or below this median indicating students who have made appropriate reading progress, to assist in assessment and to create parameters for selecting students for interventional programs who are struggling readers or at risk for developing difficulties in reading proficiency later.

The purpose of this study was to evaluate the ORF of students from the third to the fifth grades (with a 1-min fluency measure) to characterize their fluency and determine references of appropriate development in reading at the 50th percentile and those below this reference.

## Method

### Design

This is a quantitative, descriptive-explanatory study. The dependent variable is a 1-min fluency measure. The independent variable is student grade.

### General procedures and database

This study was approved by the Ethics Committee of the Faculdade de Filosofia e Ciências of Sao Paulo State University–UNESP-Campus de Marília-SP under protocol 2.550.190–CAAE 50201915.9.0000.5406.

For this study, a database of readings made available by the Investigation Learning Disabilities Laboratory (in Portuguese: Laboratório de Investigação dos Desvios da Aprendizagem–LIDA), registered by a research group of the National Counsel of Technological and Scientific Development (CNPq), called “Language, Learning, Education,” was used. All information related to the sample of students comprising our database was made available by the members of this group.

The readings database made available consists of 365 readings from elementary-school students from the third to the fifth grades in two cities in the interior of the state of São Paulo (in a medium- and a small-sized Brazilian city, Southeast Region of Brazil) from two different public school systems with the same teaching methodology. In the city of Marília-SP, there are 51 schools with regular elementary education in urban locations, in basic education, with 2221 students enrolled in the third year, 2119 students enrolled in the fourth year and 2033 students enrolled in the fifth year according to the School Census/(Instituto Nacional de Estudos e Pesquisas Educacionais Anísio Teixeira – INEP, 2018).

In the city of Garça-SP, there are 14 schools with regular elementary education in urban locations, in basic education, with 478 students enrolled in the third year, 436 students enrolled in the fourth year and 401 students enrolled in the fifth year according to the School Census/(Instituto Nacional de Estudos e Pesquisas Educacionais Anísio Teixeira – INEP, 2018). The schools were selected through convenience sampling (simple convenience sample). The students participating in the studies did not have a history of repeating grades; they were monolinguals and native speakers of Brazilian Portuguese. The data were digital recordings of participants reading the passage “The Umbrella” (text suitable for schooling levels) from the procedure “Protocol for Assessment of Reading Comprehension” (Cunha & Capellini, 2014).

Of the 365 readings, 98 were third-grade students (48.9% female), 130 were fourth-grade students (49.2% female), and 137 were fifth-grade students (51.8% female) (participants were elementary-school students ranging from 7 to 11 years old).

According to the latest results published (Instituto Nacional de Estudos e Pesquisas Educacionais Anísio Teixeira, 2015-2017) by the Socioeconomic Level Indicator (Inse) of basic education schools in Brazil, developed by the National Institute of Educational Studies and Research Anísio Teixeira (Inep), in the Basic Education Assessment Directorate (Daeb), the schools from which the analyzed data were obtained have an average Inse (absolute value 58.46 and 57.47), with an average rating (group 5).

The inclusion and exclusion criteria used by the laboratory researchers in the data collection of the reading audio bank are described. The inclusion criteria for the sample selection were as follows: informed consent form signed by the parents or guardians for the students; students with no history of neurological or psychiatric illnesses, uncorrected auditory and visual impairments, and cognitive performance within normal, according to the description at the school records and teachers’ reports. The exclusion criteria for the sample selection were the presence of genetic or neurological syndromes in the students, students who did not present a satisfactory reading domain level for the observation of the variable proposed in the study, and students who presented recording errors in their respective audio files.

### Specific instruments and procedures

The passage used was “The Umbrella” (history appropriate for the educational level) from the procedure “Reading Comprehension Assessment Protocol” (Cunha & Capellini, 2014). The choice for using this protocol occurred due to its careful assessment and development, since its issues were built from the rules for the psychometric tool development described by The Federal Council of Psychology. The Council is an official body that studies and establishes criteria and rules in Brazil for the construction of evaluation tools that ensures their accuracy and validity, and defines, as reliable procedures, those whose accuracy is understood as their level of consistency and their ability to reach the objectives for which they were built as their validity.

The protocol consists of four passages, two narratives, and two expository narratives. A medium-length (297 words) narrative passage was chosen. The choice of a passage with a narrative gender protocol occurred because the students had been more commonly exposed to such passages since childhood and throughout the education process, which would simplify the fluency evaluation and avoid the interference of any cultural issues of the passage in the reading results of the students of different schooling levels.

The choice of protocol also occurred because it presents passages that were selected to reach students from the third, fourth and fifth grades at representatively similar levels of difficulty for all school years, making it possible to apply a single passage in all school years.

Although the procedure is an instrument for assessing reading comprehension, due to the objectives of this study, only the reading recordings were used to assess fluency, while the multiple-choice questions were not applied.

The equipment used in the recordings was a Karsect microphone headset, which was unidirectional since the microphone picks up sounds with greater intensity and orients towards where it is directed, reducing the intensity of the external noise. The microphone was connected to an HP notebook with an Intel Pentium processor, 3 GB memory, and a 32-bit operating system. Recordings were made with an original HP software application and were saved as .wav files.

The collections were carried out by the researchers of the mentioned research group, following the guidelines for individual application. Each reading of the entire passage was recorded, taking an average of 5 min total for each individual recording session in spaces reserved for the researchers in the schools during class hours.

To analyze the readings on digital media, the following steps were planned and performed:

- *Step 1*: The rate was scored by listening to 365 digital recordings and assessing the WCPM scores, which was performed according to the reading error classification used by Begeny, Capellini, and Martins (2018) and by other researchers (Valencia et al., 2010). In this approach, the types of errors that are marked as WIPM are mispronounced words, words substituted with others, words omitted, words read out of order, addition or omission of word endings, and hesitation (words on which the student paused more than 3 s, after which he or she is told the word, and it is marked as incorrect. If necessary, the student is told to continue with the next word).

The following items indicate all situations that are marked as WCPM: words pronounced correctly, self-corrections, words decoded slowly but ultimately read correctly, repeated words, words mispronounced due to dialect or regional differences, and words inserted. To quantify errors, scoring rules are also proposed for certain situations: lines or multiple words omitted; when one or more lines are not read (four or more omitted words in sequence), they are not considered errors, although those words are excluded from the WCPM (such that this rule is applied whenever a student skips four or more words within a sentence). If the student skips one, two, or three consecutive words, each word should be counted as an error (WIPM). Regarding hyphenated words that can exist independently, each morpheme separated by a hyphen counts as an individual word if the two parts exist independently when the hyphen is removed, such as *“Guarda-chuva*” [Umbrella in Portuguese] (counts as two words but is only marked incorrect when the student misreads), as opposed to the word “*anglo-China*” (considered as one word, regardless of which or both are misread).

- *Step 2*: The data thus obtained were tabulated and processed with Microsoft Excel® 2010. Data were analyzed through descriptive statistics (mean, standard deviation, and percentiles). Percentiles 5, 10, 25, 50, 75, 90, and 95 were calculated for each grade. Stratifying these percentiles helps to understand the different levels of difficulty that students may present.
- *Step 3*: The reference table was adjusted for the selection of students eligible to receive reading fluency interventions or programs. For this, the minimum reference threshold was the 25th percentile, and the maximum reference limit was the 50th percentile. The reference to the 25th percentile represents an approximate limit on the minimum level of ORF that a student should present to benefit from a fluency program. This reference was developed through years of research and related interventions (Begeny et al., 2018; Field, Begeny, & Kim, 2019).

Thus, it was determined that in the present research, WCPM intervals (maximum and minimum limits) would be established to select students who were not making adequate reading progress based on the ORF standard published by Hasbrouck and Tindal (2006).

## Results

The results regarding the reading fluency assessment measure as a procedure for selecting struggling readers or at risk of developing reading difficulties (grades 3 to 5) are summarized in Tables 1 and 2.

**Table 1:** Distribution of means, standard deviation and WCPM percentiles for readings of the passage entitled “The Umbrella”

Oral reading fluency norms, grades 3–5			
	Third grade	Fourth grade	Fifth grade
	*n* = 98	*n* = 130	*n* = 137
		WCPM	
Mean	78.82	91.50	105.26
Standard deviation	26.840	27.152	27.360
Percentile 5	38	41	54
Percentile 10	42	52	66
Percentile 25	56	74	87
Percentile 50	76	94	107
Percentile 75	97	111	121
Percentile 90	114	124	140
Percentile 95	122	133	153

*WCPM* words correct per minute

**Table 2:** WCPM reference ranges according to student grade level

Grade level	Reference intervals for WCPM
Third grade	56–76
Fourth grade	74–94
Fifth grade	87–107

From the data presented in Table 1, students in the third year who read 86 or more WCPM, in the fourth year who read 104 or more WCPM, and in the fifth year who read 117 or more WCPM are considered students who are making adequate progress in reading. As shown in Table 1, the lower the student scored beneath the 25th percentile, the more difficulties with reading the student will present, and the higher the student scored above the 50th percentile, the better the student’s performance.

Considering the standards proposed by Hasbrouck and Tindal (2006, p. 639), in which students who read more than 10 WCPM above the 50th percentile present appropriate reading progress (unless there are other indicators for concern), the WCPM was established for Brazilian students (Table 2).

The reference intervals were calculated from the readings by the 365 students, considering that those who presented a WCPM score between the 25th and 50th percentiles did not make satisfactory progress in their reading fluency and taking the 25th percentile as the minimum reference limit and the 50th percentile as the maximum reference limit (Table 2). Students with WCPM scores at the 25th percentile or below are unlikely to benefit from a fluency-based intervention because they likely need assistance with decoding, phonics, and/or phonemic awareness.

## Discussion

Measures such as the number of WCPM offer numerous advantages for use in the context of ORF assessment. This measure has already been proven to be valid and is a quick and simple measure; it can be easily implemented in educators’ routines, either within the school routine or with professionals in their clinics. The reliability coefficient of this study could not be used if the test used because a single item test was used (number of words read correctly). If used as a screening measure for students at risk of reading difficulties, it should be performed by teachers from the third grade, since it is from this series that all students are expected to have passed the literacy phase and to move from the phase of learning to read to the phase of reading to learn. Consequently, within just a few hours, a teacher can evaluate their entire class because the assessment is performed quickly, which would also enable frequent assessments, which would, in turn, enable the monitoring of students’ progress in their fluency (Hasbrouck & Tindal, 2006; Rasinski, 2004; Rasinski & Young, 2017).

For reference values, the data obtained in this study served to identify students who were making adequate reading progress and those who could benefit from a fluency program. Among the academic skills considered central to reading success, fluency reveals not only its importance in assessing and screening key components but also in intervention response strategies and models for absorbing the demand encountered after the screening and early identification of reading difficulties (Kostewicz et al., 2016).

Considering the Brazilian studies on the characterization of ORF, we note that despite their small number (Andrade et al., 2019; Dellisa & Navas, 2013; Komeno et al., 2015; Moutinho, 2016; Oliveira et al., 2013; Pacheco & Santos, 2017; Peres & Mousinho, 2017), the results help to predict and compare student performance. It is necessary to advance the description of the results to create fluency references so that they can be used to screen for students with general reading difficulties, according to each region of the country. It is emphasized that due to the continental dimensions of the Brazilian national territory, there are considerable cultural and educational differences among regions.

Therefore, the method of assessing a measure of ORF in given passages can be used to assess student progress in reading fluency competence; to predict and compare students’ performance with peers or benchmarks (since their performance is compared over time) as well as conduct individual assessments; set annual goals; assess the effectiveness of intervention programs; develop standards for the class, school, and/or region; identify students at risk of dyslexia or in need of further intervention; and serve as the initial source of data collection in the response-to-intervention model (Mendonça & Martins, 2014).

### Implications

There are public policy problems that involve this issue of early identification in Brazil, as there are no projects or actions directed at absorbing the demand of learning disabilities within the school itself. This difficulty makes the implementation of a screening process for early identification more difficult, since once these students with difficulties have been identified, there is a corresponding need for interventions, such as intervention response models together with the need for a complete structural and practical change within the classroom to modify the deeply rooted tradition of “waiting to fail to take action” (Elliott et al., 2007). However, as observed in a recent program created by the Ministry of Education (More Literacy Program–PMAlfa), new ways of implementing the screening of reading difficulties and continuing teacher education to ensure that they master the methodologies for progress monitoring and evaluation of student performance are beginning to appear.

It is also important to underscore that recent research has focused on the development of instruments and materials suitable for this type of evaluation and progress-monitoring, such as passages that are appropriate for the grade level and classified according to their difficulty, that not only allow the modification of the “waiting to fail to act” tradition but also allow suitable fluency assessment applications with materials that not only accelerate but also facilitate evaluation (such as software and applications) (Alves et al., 2019). This approach also means that three passages of the same level of difficulty can be offered (as a collection of sequential passages) to the students for assessment (Martins & Capellini, 2018), with sets of three passages to be applied throughout the school year to facilitate the monitoring of student progress.

Despite its limitations, this study extended the literature (Andrade et al., 2019; Dellisa & Navas, 2013; Komeno et al., 2015; Moutinho, 2016; Oliveira et al., 2013; Pacheco & Santos, 2017; Peres & Mousinho, 2017) as part of the research movement to obtain ORF subsidiary reference data for professionals in the health-education interface. However, it is necessary to note that one limitation of this study is the number of samples used. To complement this study and other Brazilian research in this context, new research is needed that increases the number and the representativeness of the sample of Brazilian readers who struggle.

## Conclusion

From this study, it was possible to evaluate and characterize the reading fluency of Brazilian students. It was also possible to establish reference intervals for the assessment of ORF, which can be used to screen struggling readers or students at risk who present or may develop reading difficulties.

Therefore, similar research should be carried out and expanded to create measurement parameters related to ORF, which will help teachers make decisions about which paths need to be constructed or improved to assist those students who are presenting difficulty in this learning process.

## Data Availability

The datasets used and/or analyzed during the current study are available from the corresponding author on reasonable request.

## Abbreviations

CBM: Curriculum-based measurement
ORF: Oral reading fluency
WCPM: Words read correctly per minute
WIPM: Words read incorrectly per minute
PMAlfa: More Literacy Program
CNCB: Common National Curriculum Base
